# Supplementary material for: Gain-of-function mutant p53 together with ERG proto-oncogene drive prostate cancer by beta-catenin activation and pyrimidine synthesis
Source: Nat Commun. 2023 Aug 3;14:4671. doi: 10.1038/s41467-023-40352-4 (PMC10400651; doi:10.1038/s41467-023-40352-4)
Supplement: Supplementary file 1 — Reporting Summary [file 41467_2023_40352_MOESM1_ESM.pdf]

nature portfolio

Corresponding author(s): Drs. Haojie huang and Liguo Wang

Last updated by author(s): Jun 26, 2023

Reporting Summary

Nature Portfolio wishes to improve the reproducibility of the work that we publish. This form provides structure for consistency and transparency in reporting. For further information on Nature Portfolio policies, see our Editorial Policies and the Editorial Policy Checklist.

Statistics

For all statistical analyses, confirm that the following items are present in the figure legend, table legend, main text, or Methods section.

n/a	Confirmed	
☐	☒	The exact sample size (*n*) for each experimental group/condition, given as a discrete number and unit of measurement
☒	☐	A statement on whether measurements were taken from distinct samples or whether the same sample was measured repeatedly
☐	☒	The statistical test(s) used AND whether they are one- or two-sided<br>*Only common tests should be described solely by name; describe more complex techniques in the Methods section.*
☒	☐	A description of all covariates tested
☒	☐	A description of any assumptions or corrections, such as tests of normality and adjustment for multiple comparisons
☐	☒	A full description of the statistical parameters including central tendency (e.g. means) or other basic estimates (e.g. regression coefficient) AND variation (e.g. standard deviation) or associated estimates of uncertainty (e.g. confidence intervals)
☐	☒	For null hypothesis testing, the test statistic (e.g. *F*, *t*, *r*) with confidence intervals, effect sizes, degrees of freedom and *P* value noted<br>*Give P values as exact values whenever suitable.*
☒	☐	For Bayesian analysis, information on the choice of priors and Markov chain Monte Carlo settings
☒	☐	For hierarchical and complex designs, identification of the appropriate level for tests and full reporting of outcomes
☒	☐	Estimates of effect sizes (e.g. Cohen's *d*, Pearson's *r*), indicating how they were calculated

Our web collection on statistics for biologists contains articles on many of the points above.

Software and code

Policy information about availability of computer code

Data collection	UCSC genome browser (https://genome.ucsc.edu) was used to access and visualize gene tracks;<br>Odyssey Fc Imager-LI-COR Imaging to acquire the data from western blot;<br>CFX Maestro Software 2.3 to acquire data from RT-qPCR or ChIP-qPCR;<br>Soft Max Pro V5 to measure the OD values from cell growth assays and IC50 assays.
Data analysis	GraphPad Prism v8, Adobe Photoshop 2021, RSeQC package (v2.3.6), MACS2 (version 2.1.1), DESeq2 (version 1.30.1), bowtie2 (version 2.2.9), Genomic Regions Enrichment of Annotations Tool (GREAT), DAVID Bioinformatics Resources (https://david.ncifcrf.gov/).

For manuscripts utilizing custom algorithms or software that are central to the research but not yet described in published literature, software must be made available to editors and reviewers. We strongly encourage code deposition in a community repository (e.g. GitHub). See the Nature Portfolio guidelines for submitting code & software for further information.

Data

Policy information about availability of data

All manuscripts must include a data availability statement. This statement should provide the following information, where applicable:

- Accession codes, unique identifiers, or web links for publicly available datasets
- A description of any restrictions on data availability
- For clinical datasets or third party data, please ensure that the statement adheres to our policy

The RNA-seq and ChIP-seq data generated from the current study have been deposited in Gene Expression Omnibus (GEO) database with the accession number GSE184626: https://www.ncbi.nlm.nih.gov/geo/query/acc.cgi?acc=GSE184626.

Research involving human participants, their data, or biological material

Policy information about studies with human participants or human data. See also policy information about sex, gender (identity/presentation), and sexual orientation and race, ethnicity and racism.

Reporting on sex and gender	N.A.
Reporting on race, ethnicity, or other socially relevant groupings	N.A.
Population characteristics	N.A.
Recruitment	N.A.
Ethics oversight	N.A.

Note that full information on the approval of the study protocol must also be provided in the manuscript.

Field-specific reporting

Please select the one below that is the best fit for your research. If you are not sure, read the appropriate sections before making your selection.

☒ Life sciences ☐ Behavioural & social sciences ☐ Ecological, evolutionary & environmental sciences

For a reference copy of the document with all sections, see nature.com/documents/nr-reporting-summary-flat.pdf

Life sciences study design

All studies must disclose on these points even when the disclosure is negative.

Sample size	The sample size was determined based on our previous publication (PMIDs: 36274096, 34397171, 33419772, 30527665, 30057199). The sample size of patients was determined by TCGA database.
Data exclusions	No data were excluded.
Replication	For the RT-qPCR and ChIP-qPCR assays, three replicate was performed;<br>For ChIP-seq assay, two replicate was performed.<br>RNA-seq was generated from three or two replicate as indicated in the manuscript.<br>IC50 was determined by five independent replicate.<br>For cell growth assay, data was shown from five replicate. For the in vivo assay, data was collected and analyzed by the indication in manuscript.<br>For western blot, two independent technical replicate was performed to ensure the reproducibility. All the results from replicate for each experiment were consistent.<br>Others were indicated in the figure legends.
Randomization	For the RT-qPCR, ChIP-qPCR, IC50 or cell growth assay, cells were randomly divided into the indicated groups for the shRNAs infection or drug treatment. For the in vivo experiment, mice were allocated randomly for acquiring drug treatment.
Blinding	For the in vitro experiments, investigators involved in the group organization, treatment, sample collection were not blinded, while the investigators collecting data and generating output were blinded to all groups. For the in vivo experiments, investigators who were designing the organization of the groups and performing the treatment were not blinded to the groups and treatment, while other investigators collecting data such as tumor volume and tumor weight were blinded.

Reporting for specific materials, systems and methods

We require information from authors about some types of materials, experimental systems and methods used in many studies. Here, indicate whether each material, system or method listed is relevant to your study. If you are not sure if a list item applies to your research, read the appropriate section before selecting a response.

Materials & experimental systems

n/a	Involved in the study
	x Antibodies
	x Eukaryotic cell lines
x	Palaeontology and archaeology
	x Animals and other organisms
x	Clinical data
x	Dual use research of concern
x	Plants

Methods

n/a	Involved in the study
	x ChIP-seq
x	Flow cytometry
x	MRI-based neuroimaging

Antibodies

Antibodies used

anti-ERG (1:1000 in dilution, BioCare, CM421C), anti-ERG (1:5000 in dilution, Abcam, ab92513), anti-p53 (1:1000 in dilution, Santa Cruz Biotechnology, sc-126), anti-ERK2 (1:1000 in dilution, Santa Cruz, sc-1647), anti-c-Myc (1:1000 in dilution, Santa Cruz Biotechnology, sc-40), anti-cyclin D1 (1:500 in dilution, Santa Cruz Biotechnology, sc-753), anti-CK8/CK18 (1:3000 in dilution, DSHB, AB 531826), anti-SMA (1:2000 in dilution, Dako, M0851), anti-active-β-Catenin (1:1000 in dilution, Millipore, 05-665), anti-β-Catenin (1:1000 in dilution, BD Biosciences, 610153), anti-RRM1 (1:1000 in dilution, Cell Signaling Technology, #8637), anti-RRM2 (1:1000 in dilution, Cell Signaling Technology, #65939), anti-UMPS (1:1000 in dilution, NOVUS, #85896), anti-AR (1:10000 in dilution, Abcam, ab108341), anti-Ki67 (1:10000 in dilution, Abcam, ab15580), anti-CBP (1:2000 in dilution, Santa Cruz Biotechnology, sc-583), anti-LEF1 (1:1000 in dilution, Cell Signaling Technology, #2230S), anti-TCF3 (1:2000 in dilution, Proteintech, 14519-1-AP), anti-TCF4 (1:2000 in dilution, Proteintech, 22337-1-AP), anti-Histone H3 (acetyl K27) (2 µg for ChIP, Abcam, ab177178) and anti-RNA polymerase II CTD repeat YSPTSPS (phospho S2) (2 µg for ChIP, Abcam, ab5095).
Anti-mouse secondary antibody (115-035-003, Jackson Immunoresearch), 1:10000 in dilution,
Anti-mouse secondary antibody, light chain specific (115-035-174, Jackson Immunoresearch), 1:5000 in dilution,
Anti-rabbit secondary antibody (111-035-144, Jackson Immunoresearch). anti-Ki67 (ab15580, Abcam). 1:10000 in dilution.

Validation

All the antibodies were validated in the application of WB or IHC or ChIP/ChIP-seq according to their manufactures' instruction.
The commercial information for the antibodies are as following:
anti-ERG (BioCare, CM421C): https://biocare.net/product/erg-antibody/
anti-ERG (Abcam, ab92513): https://www.abcam.com/erg-antibody-epr3864-ab92513.html
anti-p53 (Santa Cruz Biotechnology, sc-126): https://datasheets.scbt.com/sc-126.pdf
anti-ERK2 (SC-1647, Santa Cruz Biotechnology): https://datasheets.scbt.com/sc-1647.pdf
anti-c-Myc (9E10) (SC-40, Santa Cruz Biotechnology): https://datasheets.scbt.com/sc-40.pdf
anti-cyclin D1 (Santa Cruz Biotechnology, sc-753): https://datasheets.scbt.com/sc-753.pdf
anti-CK8/CK18 ((DSHB, AB 531826): https://dshb.biology.uiowa.edu/TROMA-I
anti-SMA (Dako, M0851): https://www.agilent.com/en/product/immunohistochemistry/antibodies-controls/primary-antibodies/actin-(smooth-muscle)-(concentrate)-76542
anti-Active-β-Catenin (Millipore, 05-665): https://www.emdmillipore.com/US/en/product/Anti-Active-Catenin-Anti-ABC-Antibody-clone-8E7,MM_NF-05-665?ReferrerURL=https%3A%2F%2Fwww.google.com%2F&bd=1
anti-β-Catenin (BD Biosciences, 610153): https://www.bdbiosciences.com/content/dam/bdb/products/global/reagents/microscopy-imaging-reagents/immunofluorescence-reagents/610154_base/pdf/610153.pdf
anti-RRM1 (Cell signaling technology, #8637): https://www.cellsignal.com/products/primary-antibodies/rrm1-d12f12-xp-rabbit-mab/8637
anti-RRM2 (Cell signaling technology, #65939): https://www.cellsignal.com/products/primary-antibodies/rrm2-e7y9j-xp-rabbit-mab/65939
anti-UMPS (NOVUS, #85896): https://www.novusbio.com/products/umps-antibody_nbp1-85896,
anti-AR (Abcam, ab108341): https://www.abcam.com/androgen-receptor-antibody-er1792-chip-grade-ab108341.html
anti-Ki67 (Abcam, ab15580): https://www.abcam.com/ki67-antibody-ab15580.html,
anti-CBP (Santa Cruz Biotechnology, sc-583): https://datasheets.scbt.com/sc-583.pdf
anti-LEF1 (Cell signaling technology, #2230S): https://www.cellsignal.com/products/primary-antibodies/lef1-c12a5-rabbit-mab/2230
anti-TCF3 (Proteintech, 14519-1-AP): https://www.ptglab.com/products/TCF7L1-Antibody-14519-1-AP.htm
anti-TCF4 (Proteintech, 22337-1-AP): https://www.ptglab.com/products/TCF4-Antibody-22337-1-AP.htm
anti-Histone H3 (acetyl K27) (Abcam, ab177178): https://www.abcam.com/histone-h3-acetyl-k27-antibody-ep16602-chip-grade-ab177178.html
anti-RNA polymerase II CTD repeat YSPTSPS (phospho S2) (Abcam, ab5095): https://www.abcam.com/rna-polymerase-ii-ctd-repeat-ysptsps-phospho-s2-antibody-ab5095.html
Peroxidase AffiniPure Goat Anti-Mouse IgG, light chain specific: https://www.jacksonimmuno.com/catalog/products/115-035-174
Peroxidase AffiniPure Goat Anti-Mouse IgG (H+L): https://www.jacksonimmuno.com/catalog/products/115-035-003
Peroxidase AffiniPure Goat Anti-Rabbit IgG (H+L): https://www.jacksonimmuno.com/catalog/products/111-035-144

Eukaryotic cell lines

Policy information about cell lines and Sex and Gender in Research

Cell line source(s)	VCaP, DU145, LNCaP, PC-3, 22Rv1 and 293T cells were purchased from American Type Culture Collection (ATCC). C4-2 cells were purchased from Uro Corporation.
Authentication	Cell types were authenticated by morphology and/or western blot.
Mycoplasma contamination	All the cell lines were tested negative for mycoplasma contamination during the study.
Commonly misidentified lines (See ICLAC register)	No misidentified lines reported.

Animals and other research organisms

Policy information about studies involving animals; ARRIVE guidelines recommended for reporting animal research, and Sex and Gender in Research

Laboratory animals	Six-week old male SCID mice (generated in house) were used. All mice were housed in 22°C, 55% humidity on average with a 12-h light/12-h dark cycle and access to food and w
Wild animals	No Wild animals used in this study.
Reporting on sex	Only the male mice were involved in the study since the current study was focused on the prostate oncogenesis.
Field-collected samples	No field-collected samples were involved in this study.
Ethics oversight	Mice experiment was approved by the Institutional Animal Care and Use Committee (IACUC) at the Mayo Clinic.

Note that full information on the approval of the study protocol must also be provided in the manuscript.

Plants

Seed stocks	N.A.
Novel plant genotypes	N.A.
Authentication	N.A.

ChIP-seq

Data deposition

- [x] Confirm that both raw and final processed data have been deposited in a public database such as GEO.
- [x] Confirm that you have deposited or provided access to graph files (e.g. BED files) for the called peaks.

Data access links
May remain private before publication.

The RNA-seq and ChIP-seq data have been deposited in Gene Expression Omnibus (GEO) database with the accession number GSE184626: https://www.ncbi.nlm.nih.gov/geo/query/acc.cgi?acc=GSE184626.

Files in database submission

Accession	Title	Release date	Status	Supplementary files
GSE184623	TMPRSS2-ERG and gain-of-function p53 mutants co-dictate pyrimidine synthesis and prostate cancer fitness [ERG and p53 ChIP-seq]	Dec 31, 2022	approved	None
GSM5593834	VCaP_ERG-Rep1	Dec 31, 2022	approved	BW
GSM5593835	VCaP_ERG-Rep2	Dec 31, 2022	approved	BW
GSM5593836	VCaP_p53-Rep1	Dec 31, 2022	approved	BW
GSM5593837	VCaP_p53-Rep2	Dec 31, 2022	approved	BW
GSM5593838	VCaP_Input	Dec 31, 2022	approved	BW

Genome browser session (e.g. UCSC)

UCSC

Methodology

Replicates	2 biological replicated.
Sequencing depth	GSM5593834 VCaP_ERG-Rep1 24326480 unique mapped reads in total of 33322029 reads.

GSM5593835	VCaP_ERG-Rep2	22953536 unique mapped reads in total of 30229571 reads.
GSM5593836	VCaP_p53-Rep1	18407592 unique mapped reads in total of 25058409 reads.
GSM5593837	VCaP_p53-Rep2	19989314 unique mapped reads in total of 27826635 reads.
GSM5593838	VCaP_Input	65315062 unique mapped reads in total of 84284682reads.

All the data is Paired-end with 51 bp of each read.

Antibodies

ERG (ab92513, Abcam), p53 (DO-1) (sc126, Santa Cruz Biotechnology)

Peak calling parameters

The raw reads were subjected to the human reference genome (GRCh37/hg38) using bowtie2 (version 2.2.9). MACS2 (version 2.1.1) was run to perform the peak calling with a p value threshold of 1 × 10-3 for ERG ChIP-seq a p value threshold of 1 x 10-10 for p53 ChIP-seq.

Data quality

There are 14337 (FDR<0.05) peaks and 13149 (FDR<0.05, FC>5) peaks for VCaP_ERG-Rep1,
there are 11237 (FDR<0.05) peaks and 10265 (FDR<0.05, FC>5) peaks for VCaP_ERG-Rep2,
there are 50803 (FDR<0.05) peaks and 17448 (FDR<0.05, FC>5) peaks for VCaP_p53-Rep1,
there are 39374 (FDR<0.05) peaks and 7288 (FDR<0.05, FC>5) peaks for VCaP_p53-Rep2.

Software

Bowtie2 (version 2.2.9) for the row reads generation,
MACS2 (version 2.1.1) was run to perform the peak calling,
Genomic Regions Enrichment of Annotations Tool (GREAT) for the assignment of peaks to potential target genes.
