# Supplementary material for: Gain-of-function mutant p53 together with ERG proto-oncogene drive prostate cancer by beta-catenin activation and pyrimidine synthesis
Source: Nat Commun. 2023 Aug 3;14:4671. doi: 10.1038/s41467-023-40352-4 (PMC10400651; doi:10.1038/s41467-023-40352-4)
Supplement: Supplementary file 2 — Supplementary Information [file 41467_2023_40352_MOESM2_ESM.pdf]

a

	A	B	Both	A not B	B not A	Neither	p-Value	Co-occurence
(TCGA)	*ERG* fusion	*TP53* $^{WT/-}$	21	182	27	268	0.7594	No
	ERG fusion	*TP53* $^{-/-}$	13	190	8	287	0.0691	No
	ERG fusion	*TP53* $^{mutation/-}$	20	183	26	269	0.8753	No

b

	A	B	Both	A not B	B not A	Neither	p-Value	Co-occurence
(SU2C)	*ERG* fusion	*TP53* $^{WT/-}$	53	76	70	245	0.0001	Yes
	ERG fusion	*TP53* $^{-/-}$	24	120	5	295	0.0075	Yes
	ERG fusion	*TP53* $^{mutation/-}$	53	76	70	245	0.0001	Yes
	ERG fusion	*TP53* $^{DBD\ mutation/-}$	47	82	59	256	0.0001	Yes

c

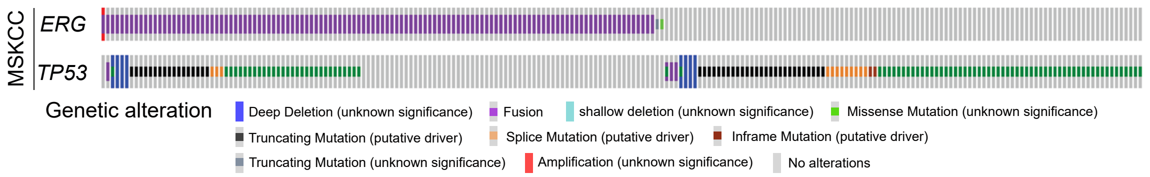

d

	A	B	Both	A not B	B not A	Neither	p-Value	Co-occurence
(MSKCC)	*ERG* fusion	*TP53* inactivation	58	70	118	255	0.0040	Yes

e

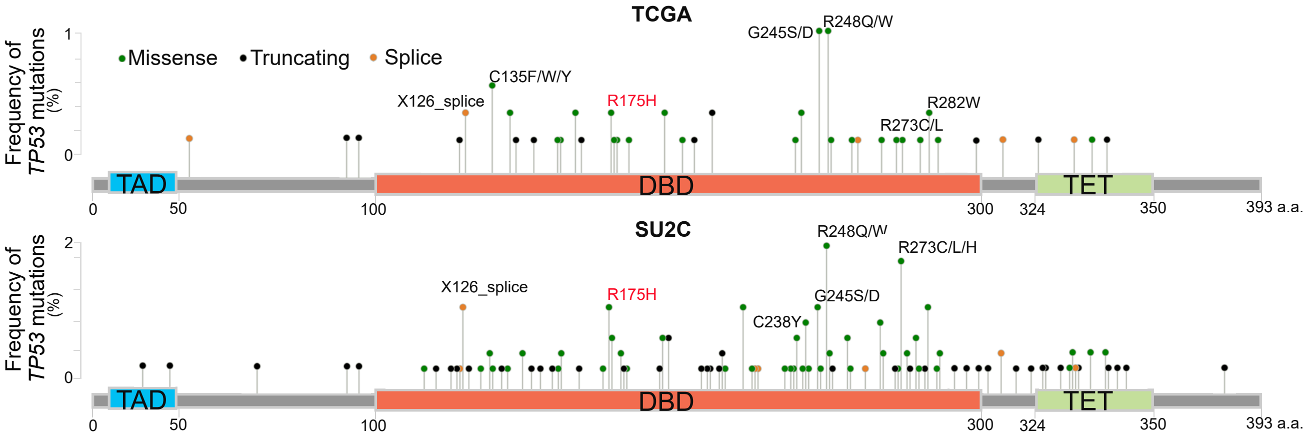

Supplementary Fig. 1. Co-occurrence of TMPRSS2-ERG and p53 alteration in PCa patient samples. **a**,**b** Fisher exact test (two-tailed) of the association between *TMPRRS2-ERG* fusion and different *TP53* alterations in PCa patient samples from the TCGA (**a**) or SU2C cohort (**b**). **c** OncoPrint image from cBioPortal showing the percentage of genetic alterations in the *ERG* and *TP53* genes in PCa patient specimens from the MSKCC cohort. **d** Fisher exact test (two-tailed) of the association between *TMPRRS2-ERG* fusion and *TP53* inactivation alterations in PCa patient samples from the MSKCC cohort. **e** *TP53* gene mutations (missense, truncating and splice) occurred in the PCa patient samples from the TCGA (upper) and SU2C (lower) cohorts.

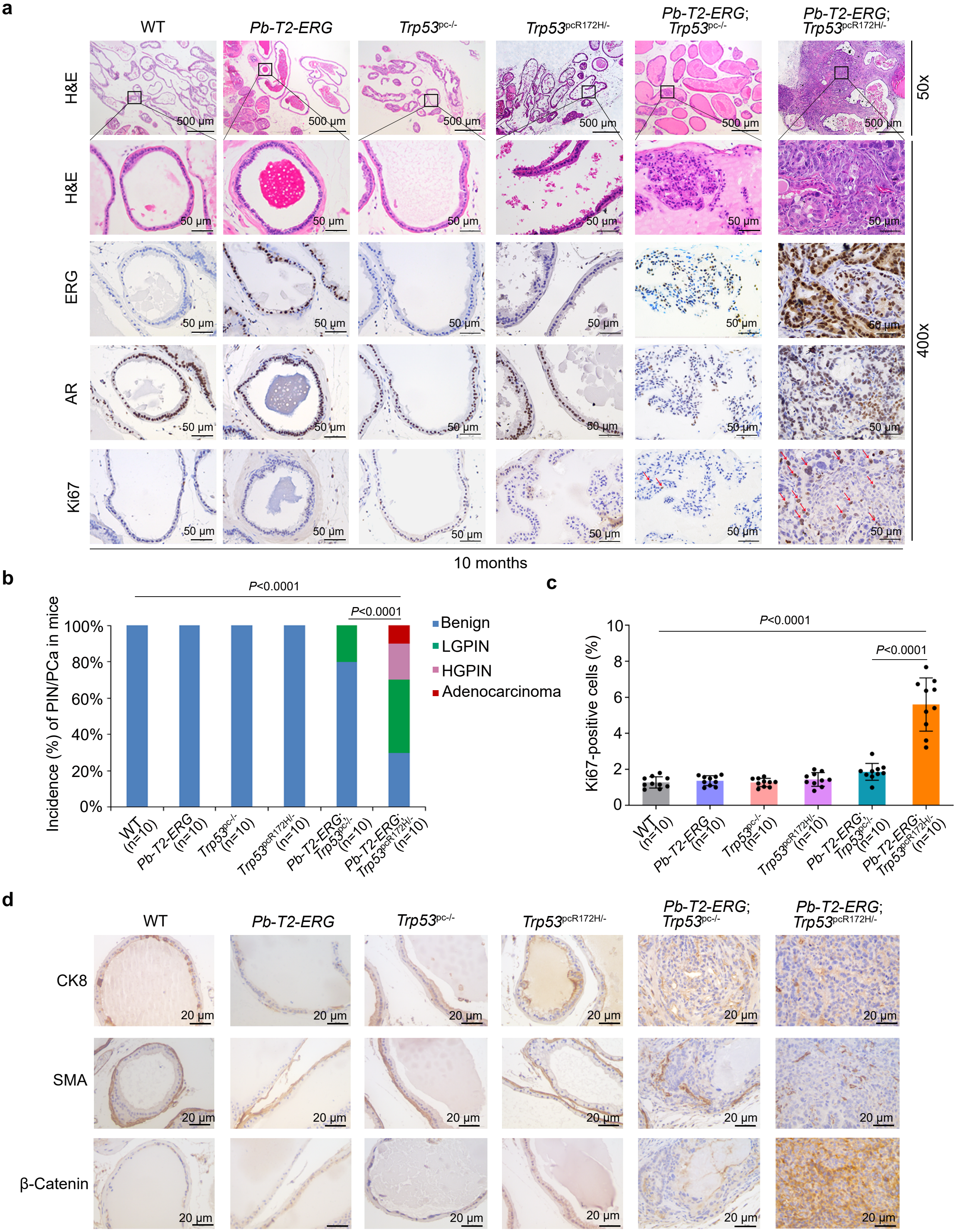

a
WT
Pb-T2-ERG
Trp53pc-/-
Trp53pcR172H/-
Pb-T2-ERG; Trp53pc-/-
Pb-T2-ERG; Trp53pcR172H/-
H&E
H&E
ERG
AR
Ki67
50x
400x
500 µm
50 µm
10 months
b
P<0.0001
P<0.0001
Incidence (%) of PIN/PCa in mice
100%
80%
60%
40%
20%
0%
Benign
LGPIN
HGPIN
Adenocarcinoma
WT (n=10)
Pb-T2-ERG (n=10)
Trp53pc-/- (n=10)
Trp53pcR172H/- (n=10)
Pb-T2-ERG; Trp53pc-/- (n=10)
Pb-T2-ERG; Trp53pcR172H/- (n=10)
c
P<0.0001
P<0.0001
Ki67-positive cells (%)
10
8
6
4
2
0
WT (n=10)
Pb-T2-ERG (n=10)
Trp53pc-/- (n=10)
Trp53pcR172H/- (n=10)
Pb-T2-ERG; Trp53pc-/- (n=10)
Pb-T2-ERG; Trp53pcR172H/- (n=10)
d
WT
Pb-T2-ERG
Trp53pc-/-
Trp53pcR172H/-
Pb-T2-ERG; Trp53pc-/-
Pb-T2-ERG; Trp53pcR172H/-
CK8
SMA
β-Catenin
20 µm

Supplementary Fig. 2. Co-expression of ERG and GOF mutant p53 induces prostate tumorigenesis in mice and IHC analysis of CK8, SMA and β-catenin expression in the mice with the indicated genotypes. a Representative images of H&E and IHC of ERG, AR and Ki67 proteins in prostate tissues from mice with the indicated genotypes at 10 months of age. **b** Quantification of incidences of PIN and/or cancer in mice with indicated genotypes shown in (**a**). **c** Quantification of Ki67 positive cells in prostate tissues from mice shown in (**a**). **d** Representative images of IHC of CK8, SMA and β-catenin proteins in prostate tissues from mice with the indicated genotypes at 15 months of age. Data in **c** was shown as means ± s.d. from indicated sample size. For each sample, five independent fields were included for the calculation. $\chi 2$ test was performed in **b**. Two-tailed Student's t test was performed in **c**.

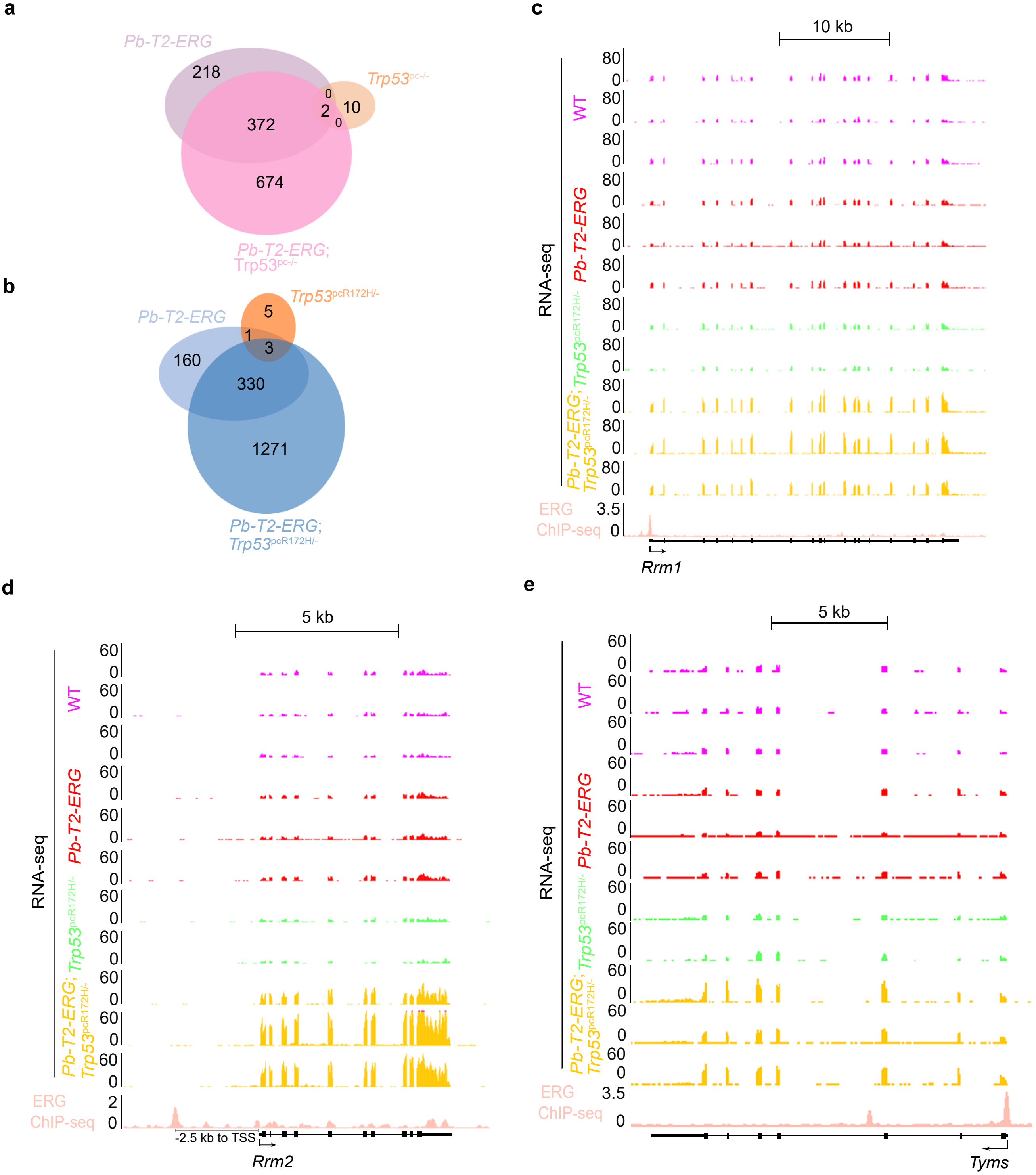
a
Pb-T2-ERG
Trp53pc-/-
Pb-T2-ERG; Trp53pc-/-
218
372
674
0
2
0
10
b
Pb-T2-ERG
Trp53pcR172H/-
Pb-T2-ERG; Trp53pcR172H/-
5
1
3
160
330
1271
c
10 kb
RNA-seq
WT
Pb-T2-ERG
Trp53pcR172H/-
Pb-T2-ERG; Trp53pcR172H/-
ERG ChIP-seq
80
0
3.5
Rrm1
d
5 kb
60
2
-2.5 kb to TSS
Rrm2
e
5 kb
Tyms

Supplementary Fig. 3. Comparison of the genes uniquely upregulated in *Pb-T2-ERG;Trp53*$^{\mathbf{pcR172H/-}}$, *Pb-T2-ERG;Trp53*$^{\mathbf{pc-/-}}$ and other genotypic mice. a,**b** Venn diagram showing the genes uniquely expressed in prostate tissues from the indicated genotypic mice compared to WT mice at 15 months of age as revealed by RNA-seq data (n=3/group except *Trp53*$^{pcR172H/-}$ group for which the data from one mouse were excluded from analysis due to poor quality). **c-e** UCSC Genome Browser screenshots showing the RNA-seq from benign or malignant prostate tissues of mice with the indicated genotypes at 15 months of age and ERG ChIP-seq (GSM1145303) data in the loci of *Rrm1* (**c**), *Rrm2* (**d**), and *Tyms* (**e**) genes.

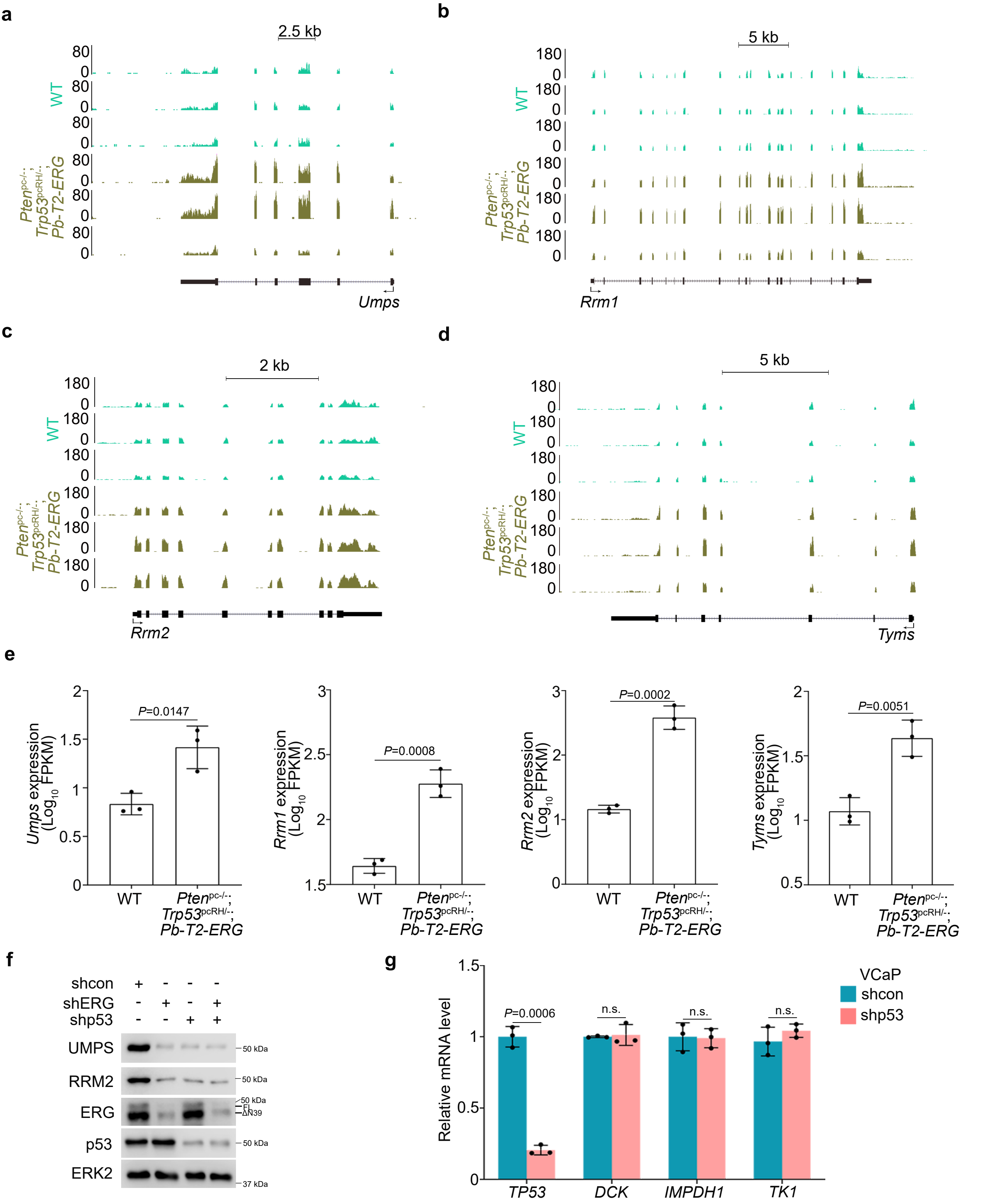

a
2.5 kb
80
0
WT
Pten pc-/-; Trp53 pcRH/-; Pb-T2-ERG
Umps
b
5 kb
180
0
Rrm1
c
2 kb
Rrm2
d
5 kb
Tyms
e
Umps expression (Log10 FPKM)
P=0.0147
Rrm1 expression (Log10 FPKM)
P=0.0008
Rrm2 expression (Log10 FPKM)
P=0.0002
Tyms expression (Log10 FPKM)
P=0.0051
f
shcon
shERG
shp53
UMPS
RRM2
ERG
p53
ERK2
50 kDa
37 kDa
FL
ΔN39
g
Relative mRNA level
P=0.0006
n.s.
TP53
DCK
IMPDH1
TK1
VCaP
shcon
shp53

Supplementary Fig. 4. Expression of PSG in *Pten*$^{pc-/-}$*;Trp53*$^{pcR172H/-}$*; Pb-T2-ERG* mice, Expression of PSG in VCaP cells after knockdown of ERG or/and p53 and other pyrimidine metabolism gene expression after knockdown of p53 in VCaP cells. a-d UCSC Genome Browser screenshots showing the RNA-seq of *Umps* (**a**), *Rrm1* (**b**), *Rrm2* (**c**) and *Tyms* (**d**) in prostate tumors from *Pten*$^{pc-/-}$*;Trp53*$^{pcR172H/-}$*;Pb-T2-ERG* mice. **e** Quantitative data showing the RNA-seq reads of PSG mRNAs in the prostate tumors from the mice with the indicated genotypes. Log_{10} (FPKM) was calculated for the expression of PSG mRNAs. Two-tailed Student's t-test was used to assess the significance. **f,g** Western blot (**f**) and RT-qPCR (**g**) analysis of the indicated proteins and PSG gene mRNAs in VCaP cells stably expressing control (shcon) or gene-specific shRNAs. Data in **e** was shown as means ± s.d. (n=3). Data in **g** was shown as means ± s.d. from three independent replicates. The western blot assay in **f** was repeated two independent times with similar results. Two-tailed Student's t-test was used in **e** and **g**.

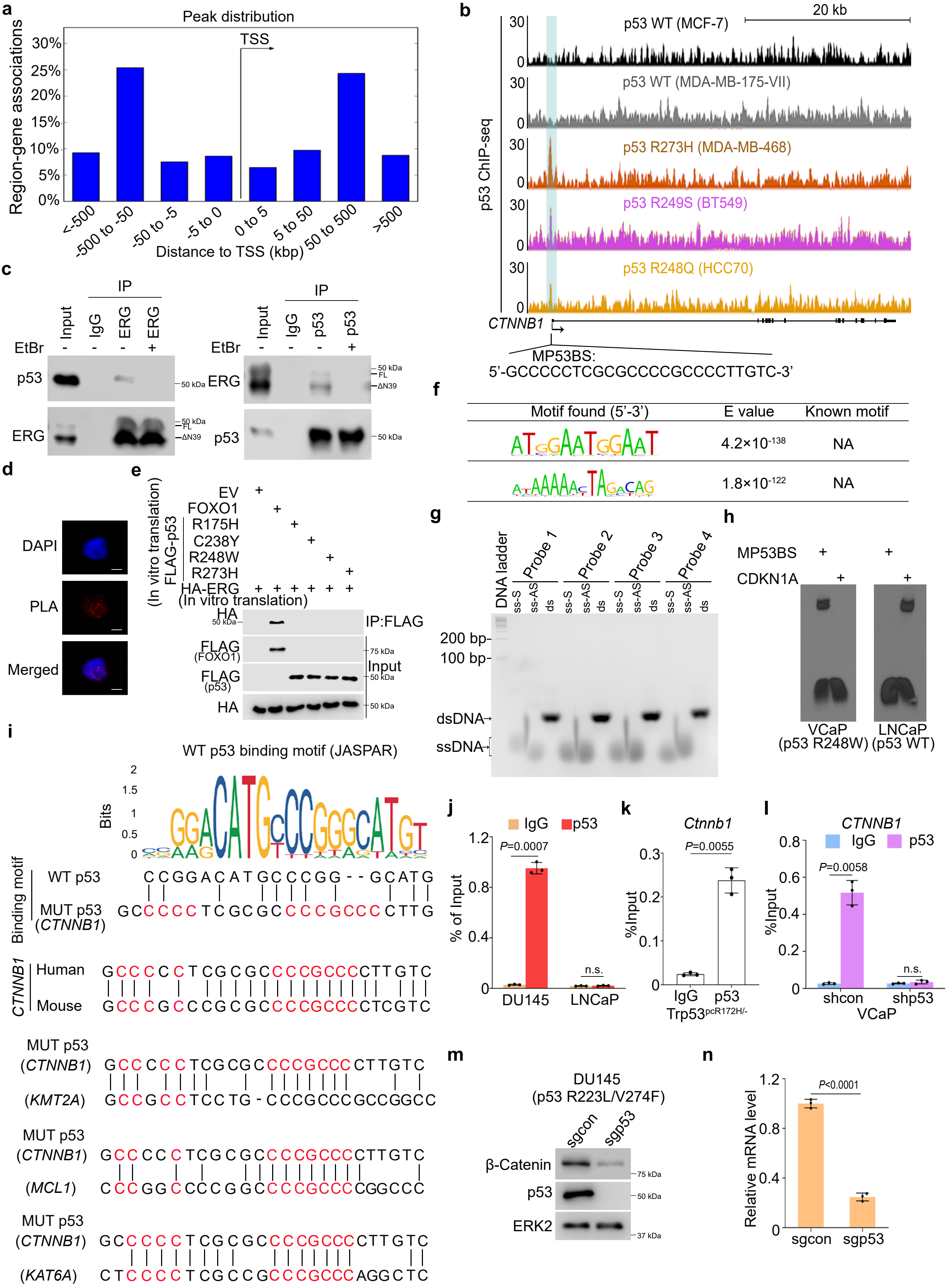
a
Peak distribution
TSS
Region-gene associations
30%
25%
20%
15%
10%
5%
0%
<-500
-500 to -50
-50 to -5
-5 to 0
0 to 5
5 to 50
50 to 500
>500
Distance to TSS (kbp)
b
20 kb
p53 ChIP-seq
p53 WT (MCF-7)
p53 WT (MDA-MB-175-VII)
p53 R273H (MDA-MB-468)
p53 R249S (BT549)
p53 R248Q (HCC70)
CTNNB1
MP53BS:
5'-GCCCCCTCGCGCCCCGCCCCTTGTC-3'
c
IP
Input
IgG
ERG
EtBr
p53
50 kDa
FL
ΔN39
d
DAPI
PLA
Merged
e
EV
FOXO1
FLAG-p53
(In vitro translation)
R175H
C238Y
R248W
R273H
HA-ERG
(In vitro translation)
HA
IP:FLAG
FLAG (FOXO1)
FLAG (p53)
Input
75 kDa
50 kDa
f
Motif found (5'-3')
E value
Known motif
4.2×10^-138
NA
1.8×10^-122
NA
g
DNA ladder
Probe 1
Probe 2
Probe 3
Probe 4
ss-S
ss-AS
ds
200 bp
100 bp
dsDNA
ssDNA
h
MP53BS
CDKN1A
VCaP (p53 R248W)
LNCaP (p53 WT)
i
WT p53 binding motif (JASPAR)
Bits
2
1.5
1
0.5
0
Binding motif
WT p53 CCGGACATGCCCGG--GCATG
MUT p53 (CTNNB1) GCCCCCTCGCGCCCCGCCCCTTG
CTNNB1
Human GCCCCCTCGCGCCCCGCCCCTTGTC
Mouse GCCCGCCCGCGCCCCGCCCCTCGTC
MUT p53 (CTNNB1) GCCCCCTCGCGCCCCGCCCCTTGTC
(KMT2A) GCCGCCTCCTG-CCCGCCCGCCGGCC
MUT p53 (CTNNB1) GCCCCCTCGCGCCCCGCCCCTTGTC
(MCL1) CCCGGCCCCGGCCCCGCCCCGGCCC
MUT p53 (CTNNB1) GCCCCCTCGCGCCCCGCCCCTTGTC
(KAT6A) CTCCCCTCGCCGCCCGCCCAGGCTC
j
IgG
p53
% of Input
P=0.0007
n.s.
DU145
LNCaP
k
Ctnnb1
P=0.0055
%Input
IgG
p53
Trp53pcR172H/-
l
CTNNB1
IgG
p53
P=0.0058
n.s.
%Input
shcon
shp53
VCaP
m
DU145
(p53 R223L/V274F)
sgcon
sgp53
β-Catenin
p53
ERK2
75 kDa
50 kDa
37 kDa
n
P<0.0001
Relative mRNA level
sgcon
sgp53

Supplementary Fig. 5. GOF mutant p53 binding of *CTNNB1* gene promoter, DNA binding motif analysis, and comparison of mutant p53 binding sequences. a, Distribution of p53 R248W-bound peaks revealed by p53 ChIP-seq in VCaP cells. **b** UCSC Genome Browser screenshots showing ChIP-seq results of p53 WT and p53 mutants from breast cancer cell lines. **c** Western blot analysis of immunoprecipitated proteins from the co-IP assay using the VCaP cell lysate with or without EtBr (50 μg/ml). **d** Representative images of proximity ligation assay (PLA) in VCaP cells. Scale bar represented 10 µm. **e** Western blot analysis of immunoprecipitated proteins from co-IP assay. In vitro translated FLAG-FOXO1 was used as a positive control. **f** MEME-ChIP DNA motif analysis in 1,116 mutant p53 (R248W)-bound ChIP-seq peaks obtained from VCaP cells. **g** Agarose gel (4%) electrophoresis of single-strand (ss) sense (S) and antisense (AS) oligos and annealed double-stranded (ds) DNA probes (#1 to #4) used for EMSA. **h** EMSA assay using indicated probes and nuclear extract from VCaP cells or LNCaP cells. **i** Alignment of MP53BS DNA sequences in human *CTNNB1* gene promoter with WT p53 binding consensus motif, the homologous sequence of MP53BS in the mouse *Ctnnb1* promoter, and the MP53BS-like sequences in targets including *KMT2A*, *MCL1* and *KAT6A*. **j** ChIP-qPCR analysis of the occupancy of mutant p53 and WT p53 at the *CTNNB1* promoter in DU145 and LNCaP cell lines, respectively. **k** ChIP-qPCR analysis of the occupancy of mutant p53 at the *Ctnnb1* promoter in *Trp53*$^{pcR172H/-}$ mouse. **l** ChIP-qPCR analysis of the occupancy of p53 mutant at the *CTNNB1* gene promoter in VCaP cells with the indicated shRNAs. **m**,**n** Western blot (**m**) and RT-qPCR (**n**) analyses of the indicated proteins and mRNAs in DU145 cells with indicated sgRNAs. Data in **j**, **k**, **l** and **n** were shown as mean ± s.d. from three

independent experiments. The western blot assays in **c**, **e** and **m** were repeated two independent times with similar results. The PLA assay in **d** was repeated two independent times with similar results. The gel electrophoresis in **g** was repeated two independent times with similar results. The EMSA assay in **h** was repeated two independent times with similar results. Two-tailed Student's t test was performed in **j**, **k**, **l** and **n**.

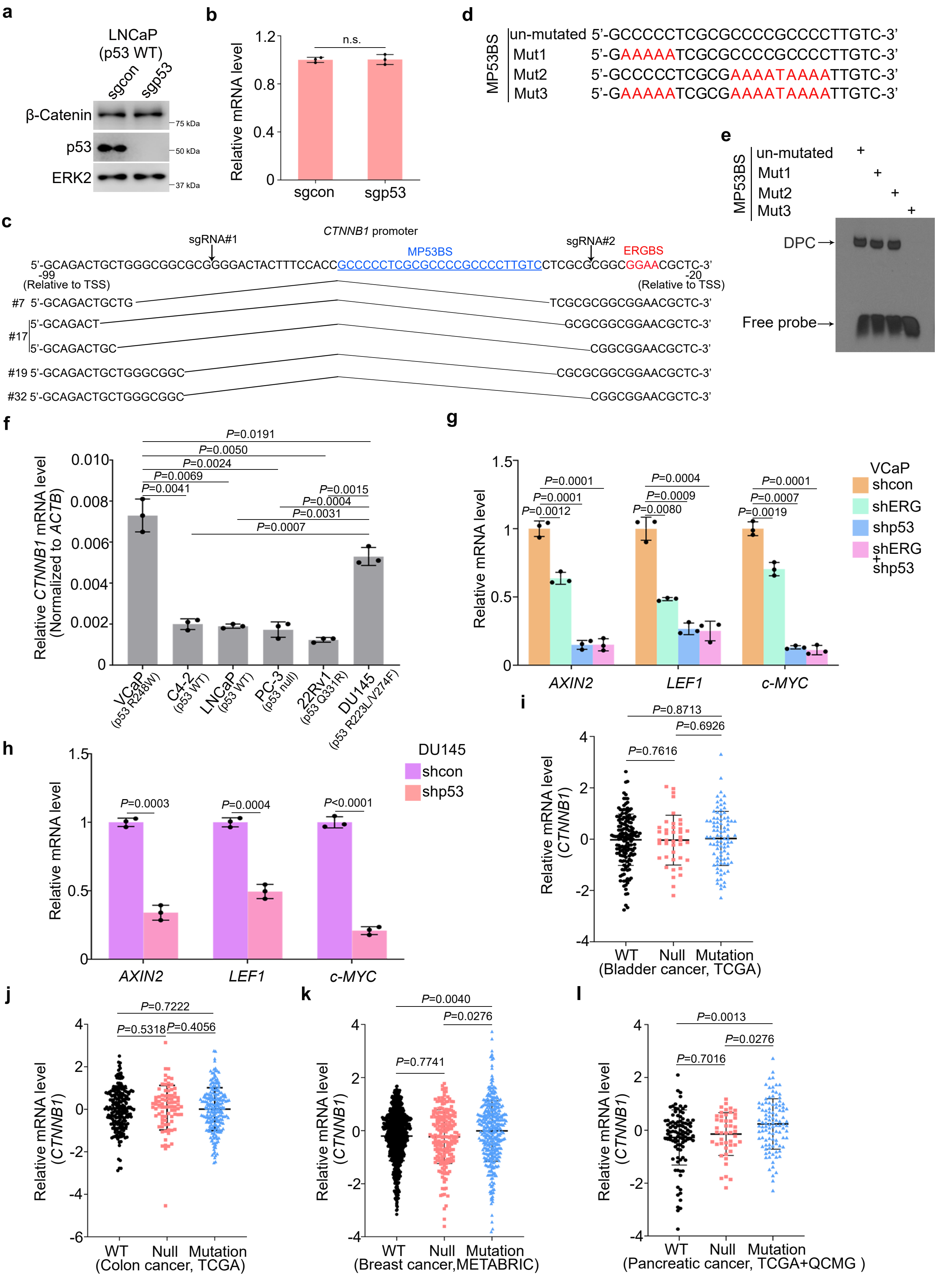
a
LNCaP (p53 WT)
sgcon
sgp53
β-Catenin
p53
ERK2
75 kDa
50 kDa
37 kDa
b
Relative mRNA level
n.s.
sgcon
sgp53
c
CTNNB1 promoter
sgRNA#1
sgRNA#2
MP53BS
ERGBS
5'-GCAGACTGCTGGGCGGCGCGGGGACTACTTTCCACCGCCCCCTCGCGCCCCGCCCCTTGTCCTCGCGCGGCGGAACGCTC-3'
-99 (Relative to TSS)
-20 (Relative to TSS)
#7 5'-GCAGACTGCTG TCGCGCGGCGGAACGCTC-3'
#17 5'-GCAGACT GCGCGGCGGAACGCTC-3'
5'-GCAGACTGC CGGCGGAACGCTC-3'
#19 5'-GCAGACTGCTGGGCGGC CGCGCGGCGGAACGCTC-3'
#32 5'-GCAGACTGCTGGGCGGC CGGCGGAACGCTC-3'
d
MP53BS
un-mutated 5'-GCCCCCTCGCGCCCCGCCCCTTGTC-3'
Mut1 5'-GAAAAATCGCGCCCCGCCCCTTGTC-3'
Mut2 5'-GCCCCCTCGCGAAAATAAAATTGTC-3'
Mut3 5'-GAAAAATCGCGAAAATAAAATTGTC-3'
e
MP53BS
un-mutated
Mut1
Mut2
Mut3
DPC
Free probe
f
Relative CTNNB1 mRNA level (Normalized to ACTB)
P=0.0191
P=0.0050
P=0.0024
P=0.0069
P=0.0041
P=0.0015
P=0.0004
P=0.0031
P=0.0007
VCaP (p53 R248W)
C4-2 (p53 WT)
LNCaP (p53 WT)
PC-3 (p53 null)
22Rv1 (p53 Q331R)
DU145 (p53 R223L/V274F)
g
Relative mRNA level
VCaP
shcon
shERG
shp53
shERG + shp53
P=0.0001
P=0.0001
P=0.0012
P=0.0004
P=0.0009
P=0.0080
P=0.0001
P=0.0007
P=0.0019
AXIN2
LEF1
c-MYC
h
Relative mRNA level
DU145
shcon
shp53
P=0.0003
P=0.0004
P<0.0001
AXIN2
LEF1
c-MYC
i
Relative mRNA level (CTNNB1)
P=0.8713
P=0.6926
P=0.7616
WT Null Mutation
(Bladder cancer, TCGA)
j
Relative mRNA level (CTNNB1)
P=0.7222
P=0.5318
P=0.4056
WT Null Mutation
(Colon cancer, TCGA)
k
Relative mRNA level (CTNNB1)
P=0.0040
P=0.0276
P=0.7741
WT Null Mutation
(Breast cancer,METABRIC)
l
Relative mRNA level (CTNNB1)
P=0.0013
P=0.0276
P=0.7016
WT Null Mutation
(Pancreatic cancer, TCGA+QCMG)

Supplementary Fig. 6. Regulation of *CTNNB1 mRNA* expression by GOF mutant p53 in human PCa cell lines and relative level of indicated mRNA in cell lines or patient.

a,**b** Western blot (**a**) and RT-qPCR (**b**) analyses of the indicated proteins and mRNAs in LNCaP cells transfected with indicated sgRNAs. n.s. not significant. **c** Top, Scheme showing the locations of MP53BS, ERGBS and two targeting sgRNAs (#1 and #2) in the *CTNNB1* gene promoter. Bottom, DNA sequence data from different clones (#7, #17, #19 and #32) with deletion (KO) of MP53BS in DU145 cells by CRISPR/Cas9. Sequence data suggest that clone #17 is a mixture of at least two different cell population with different sequences deleted. **d**,**e** MP53BS probes carrying different mutations (**d**) and nuclear extract from VCaP cells were used for EMSA assay (**e**). DPC, DNA-protein complex. **f** RT-qPCR analysis of *CTNNB1* mRNA in prostate cancer cell lines. **g** RT-qPCR analysis of the indicated mRNAs in VCaP cells expressing the indicated shRNAs. **h** RT-qPCR analysis of the indicated mRNAs in DU145 cells expressing the indicated shRNAs. **i-l** Meta-analysis of RNA-seq data showing the *CTNNB1* mRNA expression levels in the indicated genotypic subgroups of patient samples from different cancer cohort including bladder cancer (**i**), colon cancer (**j**), breast cancer (**k**), and pancreatic cancer (**l**). Data in **b**, **f**, **g** and **h** were shown as mean ± s.d. from three independent experiments. Data in **i** was shown as mean ± s.d. from WT (n=153), Null (n=41) and Mutation (n=89). Data in **j** was shown as mean ± s.d. from WT (n=211), Null (n=83) and Mutation (n=219). Data in **k** was shown as mean ± s.d. from WT (n=297), Null (n=52) and Mutation (n=108). Data in **l** was shown as mean ± s.d. from WT (n=99), Null (n=46) and Mutation (n=102). The western blot assay in **a** was repeated two independent times with similar results. The EMSA assay in **e** was repeated two

independent times with similar results. Two-tailed Student's t test was performed in **b**, **f**, **g** and **h**.

Mann-Whitey U test was used for **i**-**l**.

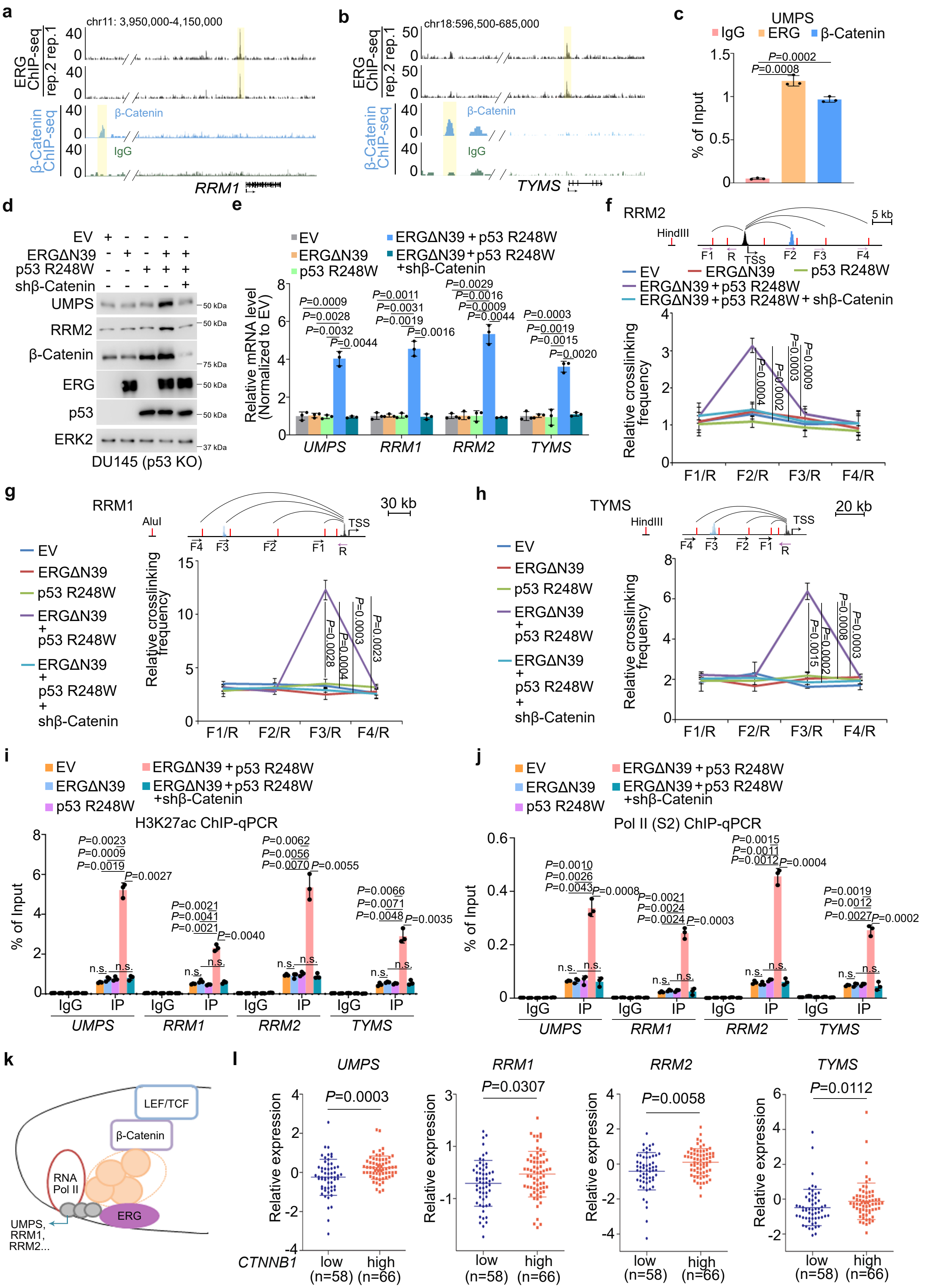
a
chr11: 3,950,000-4,150,000
ERG ChIP-seq
rep.1
rep.2
β-Catenin ChIP-seq
β-Catenin
IgG
RRM1
b
chr18:596,500-685,000
TYMS
c
UMPS
IgG ERG β-Catenin
P=0.0002
P=0.0008
% of Input
d
EV
ERGΔN39
p53 R248W
shβ-Catenin
UMPS
RRM2
β-Catenin
ERG
p53
ERK2
50 kDa
75 kDa
37 kDa
DU145 (p53 KO)
e
EV
ERGΔN39
p53 R248W
ERGΔN39 + p53 R248W
ERGΔN39 + p53 R248W +shβ-Catenin
Relative mRNA level (Normalized to EV)
UMPS RRM1 RRM2 TYMS
P=0.0009
P=0.0028
P=0.0032
P=0.0044
P=0.0011
P=0.0031
P=0.0019
P=0.0016
P=0.0029
P=0.0016
P=0.0009
P=0.0044
P=0.0003
P=0.0019
P=0.0015
P=0.0020
f
RRM2
HindIII
5 kb
TSS
F1 R F2 F3 F4
Relative crosslinking frequency
F1/R F2/R F3/R F4/R
P=0.0004
P=0.0002
P=0.0003
P=0.0009
g
RRM1
AluI
30 kb
P=0.0028
P=0.0004
P=0.0003
P=0.0023
h
TYMS
20 kb
P=0.0015
P=0.0002
P=0.0008
P=0.0003
i
H3K27ac ChIP-qPCR
% of Input
IgG IP
P=0.0023
P=0.0009
P=0.0019
P=0.0027
P=0.0021
P=0.0041
P=0.0021
P=0.0040
P=0.0062
P=0.0056
P=0.0070
P=0.0055
P=0.0066
P=0.0071
P=0.0048
P=0.0035
n.s.
j
Pol II (S2) ChIP-qPCR
P=0.0010
P=0.0026
P=0.0043
P=0.0008
P=0.0021
P=0.0024
P=0.0024
P=0.0003
P=0.0015
P=0.0011
P=0.0012
P=0.0004
P=0.0019
P=0.0012
P=0.0027
P=0.0002
k
LEF/TCF
β-Catenin
RNA Pol II
ERG
UMPS, RRM1, RRM2...
l
UMPS RRM1 RRM2 TYMS
Relative expression
P=0.0003
P=0.0307
P=0.0058
P=0.0112
CTNNB1
low (n=58)
high (n=66)

Supplementary Fig. 7. ERG and GOF mutant p53 cooperate to regulate β-catenin and PSG expression.

a,**b** UCSC Genome Browser screenshots showing the occupancy of ERG and β-catenin proteins at *RRM1* (**a**) and *TYMS* (**b**) gene loci as revealed by ChIP-seq data. **c** ChIP-qPCR analysis of ERG and β-Catenin co-occupancy at the *UMPS* gene promoter. **d,e** Western blot (**d**) and RT-qPCR (**e**) analysis of the indicated proteins and mRNAs in p53-KO DU145 cells expressing the indicated plasmids and/or shRNAs. **f-h** Chromosome Conformation Capture (3C) assay for analysis of chromatin interaction between the ERG- and β-catenin-occupied sites in the *RRM2* (**f**), *RRM1* (**g**) and *TYMS* (**h**) loci in p53-KO DU145 cells expressing the indicated plasmids and/or shRNAs. **i,j** ChIP-qPCR analysis of the levels of H3K27ac (**i**) and Pol II-S2-p (**j**) at the indicated PSG loci in p53-KO DU145 cells transfected with the indicated plasmids and/or infected lentivirus expressing the indicted shRNAs. n.s., not significant. **k** A hypothetical model depicting the probable spacial interaction of chromatin between ERG- and β-Catenin binding regions at the PSG loci. **l** Meta-analysis of RNA-seq data showing mRNA levels of *UMPS, RRM1*, *RRM2* and *TYMS* in the indicated genotypic subgroups of patient samples from the SU2C cohort. Data in **c**, **e**, **f**, **g**, **h**, **i** and **j** were shown as mean ± s.d. from three independent experiments. The western blot assay in **d** was repeated two independent times with similar results. Data in **l** was shown as mean ± s.d. from indicated samples. Two-tailed Student's t test was performed in **c**, **e**, **f**, **g**, **h**, **i** and **j**. Mann-Whitey U test was used for **l**.

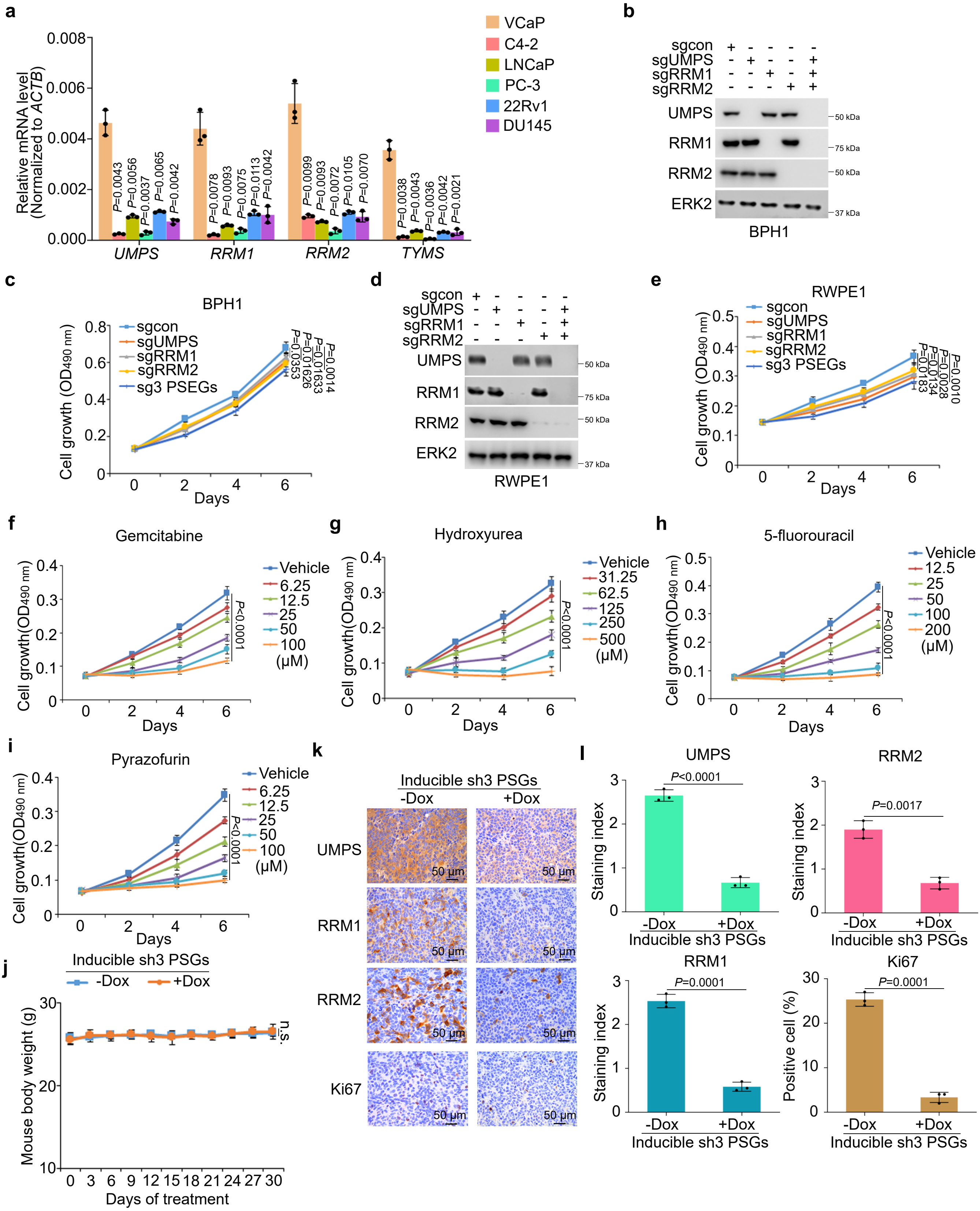

a
Relative mRNA level (Normalized to ACTB)
VCaP
C4-2
LNCaP
PC-3
22Rv1
DU145
UMPS
RRM1
RRM2
TYMS
b
sgcon
sgUMPS
sgRRM1
sgRRM2
UMPS
RRM1
RRM2
ERK2
BPH1
c
BPH1
Cell growth (OD490 nm)
sg3 PSEGs
Days
d
RWPE1
e
RWPE1
f
Gemcitabine
Vehicle
(μM)
g
Hydroxyurea
h
5-fluorouracil
i
Pyrazofurin
j
Inducible sh3 PSGs
-Dox
+Dox
Mouse body weight (g)
Days of treatment
n.s.
k
Inducible sh3 PSGs
Ki67
50 μm
l
Staining index
Positive cell (%)

Supplementary Fig. 8. **Regulation of PSGs by ERG and p53 mutant, the inhibition of VCP by different inhibitors and the effect of PSGs on PCa cell growth.**

a RT-qPCR analysis of the indicated PSG mRNAs in PCa cell lines. **b** Western blot analysis of the indicated proteins in BPH1 cells expressing the indicated sgRNAs. **c** MTS assay in BPH1 cells transduced with the indicated sgRNA as (**b**). **d** Western blot analysis of the indicated proteins in RWPE1 cells expressing the indicated sgRNAs. **e** MTS assay in RWPE1 cells transduced with the indicated sgRNA as (**d**). **f-i** MTS assay in VCaP cells treated with vehicle or different doses of gemcitabine (**f**), hydroxyurea (**g**), 5-fluorouracil (**h**) and pyrazofurin (**i**), respectively. **j** Body weight of mice treated with vehicle or Dox to induce the depletion of UMPS, RRM1 and RRM2 PSG enzymes. Data shown as mean ± s.d. (n=6 mice/group). n.s., not significant. **k** Representative images of IHC of the indicated proteins in tumors from mice treated with or without Dox. **l** Quantification of IHC staining of the indicated proteins. See details about the staining scoring and index in Methods. Data in **a** was shown as mean ± s.d. from three independent experiments. The western blot assays in **b** and **d** were repeated two independent times with similar results. Data in **c**, **e**, **f**, **g**, **h** and **i** were shown as mean ± s.d. from five replicates. Data **j** was shown as mean ± s.d. from six xenografts. Data in l was shown as mean ± s.d. from three independent samples. For each sample, five independent fields were enrolled for the calculation. Two-tailed Student's t test was performed in **a** and **l**. Two-way ANOVA was performed in **c**, **e**, **f**, **g**, **h**, **i** and **j**.

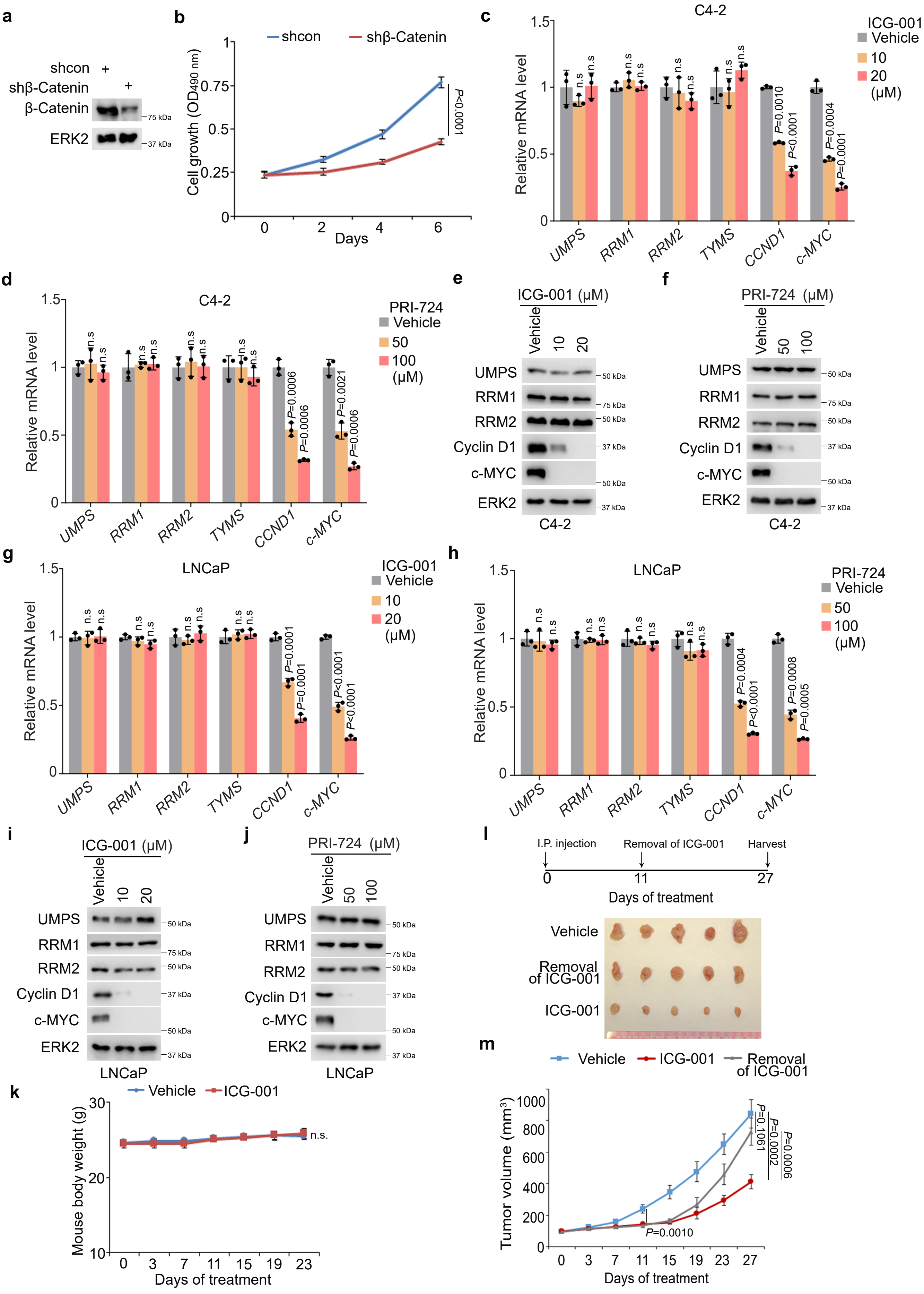
a
shcon
shβ-Catenin
β-Catenin
ERK2
75 kDa
37 kDa
b
shcon
shβ-Catenin
Cell growth (OD490 nm)
Days
P<0.0001
c
C4-2
ICG-001
Vehicle
10
20
(μM)
Relative mRNA level
UMPS
RRM1
RRM2
TYMS
CCND1
c-MYC
P=0.0010
P<0.0001
P=0.0004
P=0.0001
d
C4-2
PRI-724
Vehicle
50
100
(μM)
P=0.0006
P=0.0006
P=0.0021
P=0.0006
e
ICG-001 (μM)
Vehicle
UMPS
RRM1
RRM2
Cyclin D1
c-MYC
ERK2
C4-2
f
PRI-724 (μM)
g
LNCaP
P=0.0001
P=0.0001
P<0.0001
P<0.0001
h
LNCaP
P=0.0004
P<0.0001
P=0.0008
P=0.0005
i
ICG-001 (μM)
LNCaP
j
PRI-724 (μM)
LNCaP
k
Vehicle
ICG-001
Mouse body weight (g)
n.s.
Days of treatment
l
I.P. injection
Removal of ICG-001
Harvest
Days of treatment
Vehicle
Removal of ICG-001
ICG-001
m
Vehicle
ICG-001
Removal of ICG-001
Tumor volume (mm3)
P=0.1061
P=0.0002
P=0.0006
P=0.0010
Days of treatment

Supplementary Fig. 9. **Effects of β-Catenin inhibition on growth of PCa cells.**

a,b Western blot analysis of the indicated proteins (**a**) and MTS assay (**b**) in VCaP cells infected lentivirus expressing control (shcon) or β-Catenin-specific shRNAs. ERK2 was used as a loading control. **c,d** RT-qPCR analysis of expression of the indicated mRNAs in C4-2 cells treated with vehicle or different doses of ICG-001 (**c**) or PRI-724 (**d**). **e,f** Western blot analysis of the indicated proteins in C4-2 cells treated with vehicle or different doses of ICG-001 (**e**) or PRI-724 (**f**). **g,h** RT-qPCR analysis of expression of the indicated mRNAs in LNCaP cells treated with vehicle or different doses of ICG-001 (**g**) or PRI-724 (**h**). **i,j** Western blot analysis of the indicated proteins in LNCaP cells treated with vehicle or different doses of ICG-001 (**i**) or PRI-724 (**j**). **k** Body weight of mice during the treatment of vehicle or ICG-001. n.s., not significant. **l,m** Representative images of VCaP xenograft tumors at 27 days (**l**) or growth curves (**m**) of tumors after treatment with vehicle, ICG-001 or removal of the treatment at the indicated time points. n.s., not significant. The western blot assays in **a**, **e**, **f**, **i** and **j** were repeated two independent times with similar results. Data in **b** was shown as mean ± s.d. from five replicates. Data in **c**, **d**, **g** and **h** were shown as mean ± s.d. from three independent experiments. Data in **k** and **m** were shown as mean ± s.d. from five xenografts. Two-way ANOVA was performed in **b**, **k** and **m**. Two-tailed Student's t test was performed in **c**, **d**, **g** and **h**.

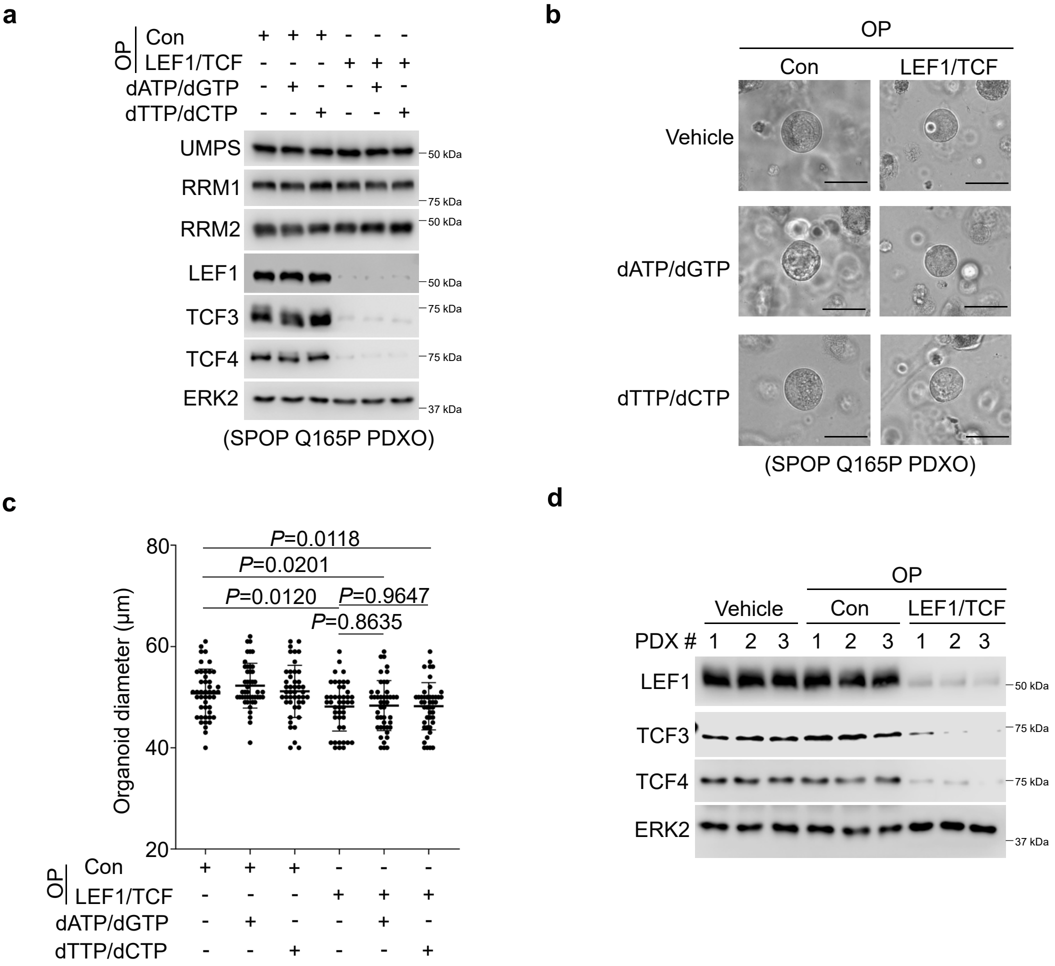
a
OP
Con
LEF1/TCF
dATP/dGTP
dTTP/dCTP
UMPS
RRM1
RRM2
LEF1
TCF3
TCF4
ERK2
50 kDa
75 kDa
50 kDa
50 kDa
75 kDa
75 kDa
37 kDa
(SPOP Q165P PDXO)
b
OP
Con
LEF1/TCF
Vehicle
dATP/dGTP
dTTP/dCTP
(SPOP Q165P PDXO)
c
P=0.0118
P=0.0201
P=0.0120
P=0.9647
P=0.8635
Organoid diameter (μm)
80
60
40
20
OP
Con
LEF1/TCF
dATP/dGTP
dTTP/dCTP
d
OP
Vehicle
Con
LEF1/TCF
PDX # 1 2 3 1 2 3 1 2 3
LEF1
TCF3
TCF4
ERK2
50 kDa
75 kDa
75 kDa
37 kDa

Supplementary Fig. 10. The effects of LEF1/TCF O'PROTAC on growth of SPOP Q165P and LuCaP23.1 PDXOs.

a-c Western blot analysis of the indicated proteins in lysate of SPOP Q165P PDXOs treated with the indicated OP and/or deoxynucleotides for 48 h (**a**) or for 3 days followed by photographing (**b**) and quantification of the diameters of organoids (**c**). Scale bar represented 100 µm. **d** Western blot analysis of the indicated proteins in PDX tumors obtained from mice with the indicated treatments (n=3 tumors/group). ERK2 was used as a loading control. The western blot assays in **a** and **d** were repeated two independent times with similar results. Data in **c** was shown as mean ± s.d. from a total of 45 organoids randomly obtained from three independent experiments. Two-tailed Students' t test was performed in **c**.
