# Supplementary material for: Gain-of-function mutant p53 together with ERG proto-oncogene drive prostate cancer by beta-catenin activation and pyrimidine synthesis
Source: Nat Commun. 2023 Aug 3;14:4671. doi: 10.1038/s41467-023-40352-4 (PMC10400651; doi:10.1038/s41467-023-40352-4)
Supplement: Supplementary file 4 — Description of Additional Supplementary Files [file 41467_2023_40352_MOESM4_ESM.pdf]

Description of Additional Supplementary Files

Supplementary Data 1
Description: The list of 901 genes uniquely upregulated in tumors from Pb-T2- ERG;Trp53 pcR172H/- mice compared to those upregulated in Pb-T2-ERG;Trp53 pc-/- counterparts.

Supplementary Data 2
Description: The list of 501 ERG target genes highly upregulated in prostate tumors from Pb-T2-ERG;Trp53 pcR172H/-mice.

Supplementary Data 3
Description: p53 mutant R248W-bound target genes identified by ChIP-seq in VCaP PCa cell line.

Supplementary Data 4
Description: MP53BS sequences found in mutant p53 R248W bound gene loci that are similar to the MP53BS (25 bp) in the CNTTB1 promoter (at least 15 out of 25 bps are matched).

Supplementary Data 5
Description: Oligonucleotide sequences of primers, shRNAs, sgRNAs and EMSA probes.
